# Functional analysis of human brain endothelium using a microfluidic device integrating a cell culture insert

**DOI:** 10.1063/5.0085564

**Published:** 2022-03-09

**Authors:** Shigenori Miura, Yuya Morimoto, Tomomi Furihata, Shoji Takeuchi

**Affiliations:** 1 Institute of Industrial Science, The University of Tokyo, Tokyo, Japan; 2 Department of Mechano-Informatics, Graduate School of Information Science and Technology, The University of Tokyo, Tokyo, Japan; 3 Laboratory of Clinical Pharmacy and Experimental Therapeutics, School of Pharmacy, Tokyo University of Pharmacy and Life Sciences, Tokyo, Japan; 4 International Research Center for Neurointelligence (WPI-IRCN), The University of Tokyo Institutes for Advanced Study (UTIAS), The University of Tokyo, Tokyo, Japan

## Abstract

The blood-brain barrier (BBB) is a specialized brain endothelial barrier structure that regulates the highly selective transport of molecules under continuous blood flow. Recently, various types of BBB-on-chip models have been developed to mimic the microenvironmental cues that regulate the human BBB drug transport. However, technical difficulties in complex microfluidic systems limit their accessibility. Here, we propose a simple and easy-to-handle microfluidic device integrated with a cell culture insert to investigate the functional regulation of the human BBB endothelium in response to fluid shear stress (FSS). Using currently established immortalized human brain microvascular endothelial cells (HBMEC/ci18), we formed a BBB endothelial barrier without the substantial loss of barrier tightness under the relatively low range of FSS (0.1–1 dyn/cm^2^). Expression levels of key BBB transporters and receptors in the HBMEC/ci18 cells were dynamically changed in response to the FSS, and the effect of FSS reached a plateau around 1 dyn/cm^2^. Similar responses were observed in the primary HBMECs. Taking advantage of the detachable cell culture insert from the device, the drug efflux activity of P-glycoprotein (P-gp) was analyzed by the bidirectional permeability assay after the perfusion culture of cells. The data revealed that the FSS-stimulated BBB endothelium exhibited the 1.9-fold higher P-gp activity than that of the static culture control. Our microfluidic system coupling with the transwell model provides a functional human BBB endothelium with secured transporter activity, which is useful to investigate the bidirectional transport of drugs and its regulation by FSS.

## INTRODUCTION

The blood-brain barrier (BBB) is a highly selective endothelial barrier, which ensures the separation of circulating blood from the brain parenchyma.^1,2^ Brain microvascular endothelial cells (BMECs) lining the cerebral capillaries express several tight junctions, adherence junctions, and key transporters/receptors, including zonula occludins-1 (ZO-1), claudin-5, CD31, vascular endothelial-cadherin (VE-cadherin), P-glycoprotein (P-gp), breast cancer resistant protein (BCRP), glucose transporter-1 (GLUT-1), and transferrin receptor (TfR). These BBB-related proteins govern the barrier tightness and the controlled transport of nutrients, metabolites, and drugs between blood and the brain.^3^ Although the essential properties of the BBB are manifested by the BMECs, the integrity and function of the BBB is regulated by several environmental factors including the cell–cell interaction and the signaling cues from the perivascular cells such as the pericytes, astrocytes, and neural cells, as well as the fluid shear stress (FSS) exerted by the blood flow.^4^

Most widely used *in vitro* BBB model is based on the two compartment transwell model, in which BMECs are cultured as a monolayer on a permeable membrane.^5^ In most cases, BMECs are co-cultured with astrocytes and pericytes in the basolateral compartment to recapitulate the paracrine or cell–cell contact communication in the brain, which usually results in the improved BBB tightness and functionality as compared to the monoculture method.^6^ This model allows for moderate throughput, easy handling for culture and functional validation but suffers from inadequate physiological relevance due to the simplicity of the model.^7^

Current advances in micro/nano fabrication and microfluidic technology enabled us to control the cellular microenvironment,^8^ including the tissue geometry and fluid dynamics^9,10^ to assess or reproduce the pathophysiological responses of the BBB to stimuli.^11^ The utility of this technology, so-called “organ-on-a-chip”^12,13^ technology, is demonstrated by modeling different aspects of the BBB, including luminal perfusion, real-time transendothelial electrical resistance (TEER) monitoring,^14^ and neurovascular coupling,^15,16^ with the increased physiological accuracy. However, the structural complexity of the BBB chips with multiple types of BBB-associated cells and three-dimensional extracellular matrices makes it difficult to analyze the bidirectional drug transport and its regulation by microenvironmental cues including FSS.

Here, we fabricated a simple and easy-to-handle microfluidic device integrating a cell culture insert to investigate the directional BBB transport and its regulation by FSS. The microfluidic device is designed to be perfusable by integrating a cell culture insert with collagen vitrigel membrane. By culturing recently established immortalized human brain microvascular endothelial cells (HBMEC/ci18 cells), which has been shown to have broad dynamic range of the compound permeability profiles,^17^ in the microfluidic device, we demonstrate that our device provides the functional human BBB endothelium model with higher activity of P-gp drug efflux pump even in the absence of BBB-associated cells.

## RESULTS AND DISCUSSION

### Perfusion culture system of the human BBB endothelium

**FIG. 1.**
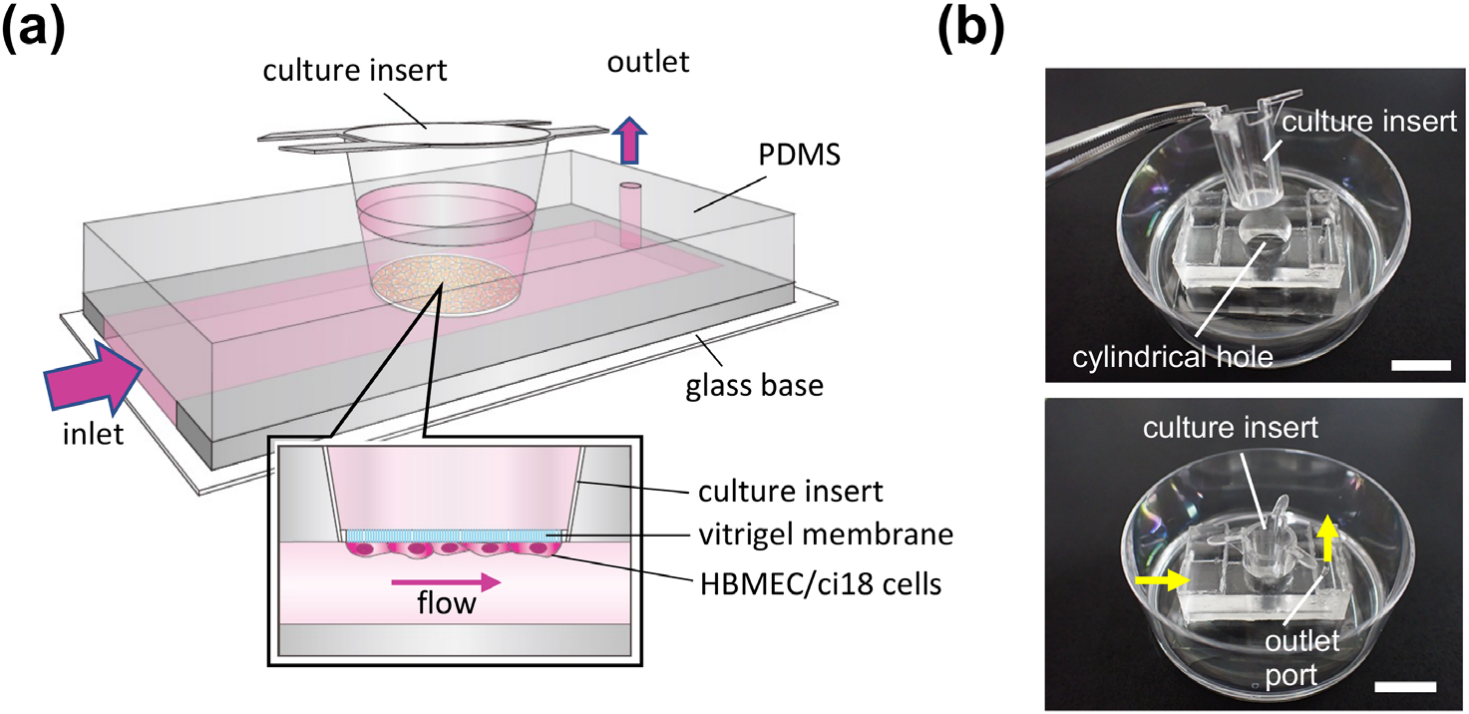
Microfluidic device for perfusion culture of human brain endothelium. (a) Schematic illustration of a microfluidic device. Cylindrical hole for a culture insert is located above the PDMS microchannel so that the vitrigel membrane of the insert and the top wall of the microchannel make a flat surface when the insert is placed into the cylindrical hole. (b) Photographic images of the fabricated microfluidic device before and after integrating a culture insert. Arrows show the direction of the medium flow. Scale bars, 10 mm.

To examine the effect of FSS on the barrier function of human brain endothelial cells, we fabricated the polydimethylsiloxane (PDMS) microfluidic device that can apply FSS to the cells grown on the bottom side of the permeable membrane of the culture insert [Fig. 1(a)], as previously described.^18^ The device simply consists of a microfluidic channel (7.8 mm in width × 19.7 mm in length × 0.15 mm in height) and a cylindrical hole located above the channel [Fig. 1(b)]. The microfluidic channel is designed to be perfusable when the culture insert is incorporated into the cylindrical hole of the device. Among the various types of culture insert, the 24-well culture insert with a collagen vitrigel membrane (a vitrified membrane of bovine native collagen type I^19^) was chosen, because this membrane is permeable enough to perform the permeability test of drugs and, unlike the porous membrane usually used for the permeability studies, transparent to observe the cell morphology on the membrane. For the perfusion culture, the PDMS device was immobilized on the culture dish, and the culture medium was circulated using a peristaltic pump (supplementary material Fig. S1). Streamlines and the flow speeds under the culture insert were almost parallel and equal (supplementary material Fig. S2).

**FIG. 2.**
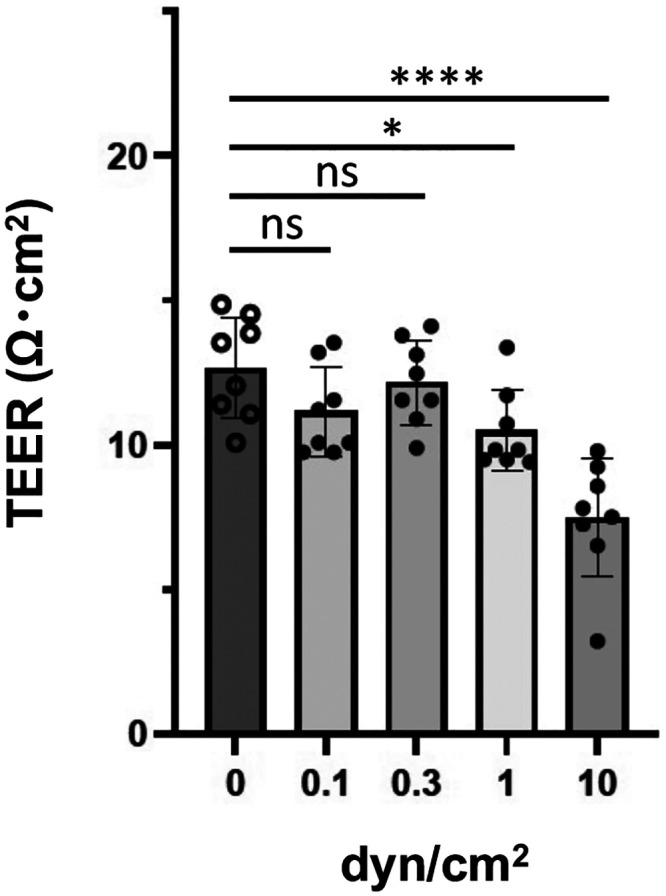
TEER measurement of HBMEC/ci18 cells cultured under flow conditions. HBMEC/ci18 cells were grown to confluence on the bottom side of the vitrigel membrane of the insert and then mounted in the microfluidic device. The cells were cultured for 3 days in the device with (0.1, 0.3, 1, and 10 dyn/cm^2^) or without medium perfusion. Data shown are the means ± S.D. (*n *=* *8). Significance was assessed by one-way ANOVA with Dunnett's multiple comparison test; ^*^P <* *0.05, ^****^P <* *0.0001.

### Formation of the human BBB endothelial barrier under the flow condition

Unlike the primary human BMECs, immortalized human BMECs are highly available, and the cellular property is generally stable. In this study, HBMEC/ci18 cells, the immortalized human BBB endothelial cells, were chosen to form human BBB endothelium, because this cell line has been shown to stably express a variety of BBB transporters and receptors and to have the broad dynamic range of the compound permeability profiles.^17^ The cells were first cultured to confluence on the bottom side of the vitrigel insert in the 24-well plate, and then the insert was carefully incorporated into the cylindrical hole of the device. The culture medium was switched from the endothelial growth medium to the VEGF-A/EGF-free medium during the perfusion culture in the device. A confluent HBMEC/ci18 cell layer was cultured under the various FSS conditions (0, 0.1, 0.3, 1, and 3 dyn/cm^2^) for 3 days in the device, and the TEER across the cell layer was measured to evaluate the barrier tightness. As shown in Fig. 2, the TEER value was 12.7 ± 1.8 Ω cm^2^ for the static control and did not significantly change at the flow rate of 0.1 and 0.3 dyn/cm^2^ (11.1 ± 1.5 Ω cm^2^ at 0.1 dyn/cm^2^; 12.2 ± 1.5 Ω cm^2^ at 0.3 dyn/cm^2^). At the flow rates of 1 and 10 dyn/cm^2^, the TEER values were decreased to 82.5% (10.5 ± 1.4 Ω cm^2^) and 59.1% (7.5 ± 2.0 Ω cm^2^) of the static control, respectively. These results suggest that, at the flow rates lower than 1 dyn/cm^2^, HBMEC/ci18 endothelial barrier can be cultured without severe loss of barrier tightness in our microfluidic device. Unlike the TEER values observed in the human iPS cells-derived brain microvascular cells or *in vivo* (estimated to over 1000 Ω cm^2^),^20–22^ the TEER values obtained in this study are considerably lower. Nevertheless, primary human BMECs^23^ and a variety of currently available and well-characterized immortalized cell line,^24^ including hCMEC/D3,^25^ hBMEC,^26^ TY10,^27^ and BB19,^28^ are reported to have similar range of TEER values to that of the HBMEC/ci18, indicating that this range of TEER values is acceptable as the BBB endothelium monoculture model.

### Effects of FSS on the expression of BBB-related genes

**FIG. 3.**
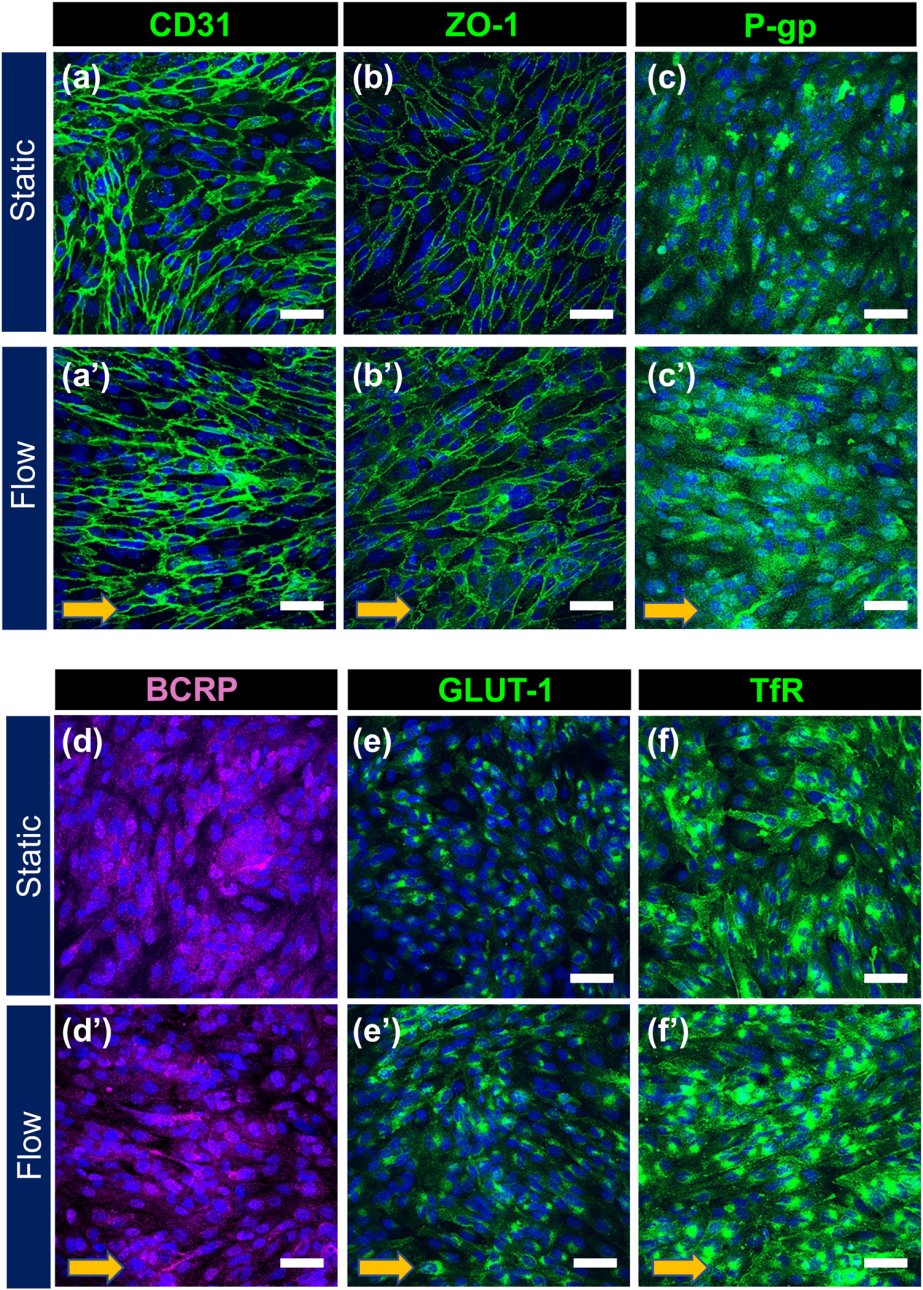
Expression of BBB marker proteins in HBMEC/ci18 cells after static and perfusion culture in the device. Confluent HBMEC/ci18 cells were cultured in the device for 72 h under static (a)–(f) or flow conditions [(a′)–(f′), 0.3 dyn/cm^2^]. Localization of BBB marker protein was analyzed by immunocytochemistry. Cell nuclei were counterstained with DAPI (*blue*). Arrows in panels (a′)–(f′) represent the flow direction. Scale bars, 50* μ*m.

It has been well known that the vascular endothelial cells in the BBB express a kind of specific protein related to the barrier tightness and permeability:^3,29,30^ endothelial cell-cell adhesion proteins (CD31, VE-cadherin), tight junction proteins (ZO-1, Claudin-5), drug efflux pumps (P-gp,^31^ BCRP^32^), GLUT-1,^33^ and TfR^34^ to uptake iron. To examine whether FSS affects the expression and localization of BBB marker proteins in HBMEC/ci18 cells, confluent HBMEC/ci18 cells cultured under the static or flow conditions for 72 h were analyzed by immunostaining (Fig. 3). Perfusion culture was performed at the flow rate of 0.3 dyn/cm^2^, because this flow rate had no significant effect on the barrier tightness in terms of TEER values (Fig. 2). As expected, no remarkable change was observed in the staining pattern and signal intensity for CD31 and ZO-1 between the static and flow conditions. The CD31 staining showed that the cells cultured under the flow condition became more narrow-shaped and to some extent tended to align along the flow direction [Figs. 3(a′) and 3(b′)] as compared to those under the static control. These morphological changes are characteristics to the FSS-stimulated endothelial cells, consistent with the fluidic response of human umbilical vein endothelial cells demonstrated in our previous report.^18^ Interestingly, the cells cultured under the flow conditions exhibited more intensive signals for P-gp, GLUT-1, and TfR [Figs. 3(c′), 3(e′), and 3(f′)] than those of the static control, while BCRP expression levels were faint and similar in both of the culture conditions [Figs. 3(d) and 3(d′)].

**FIG. 4.**
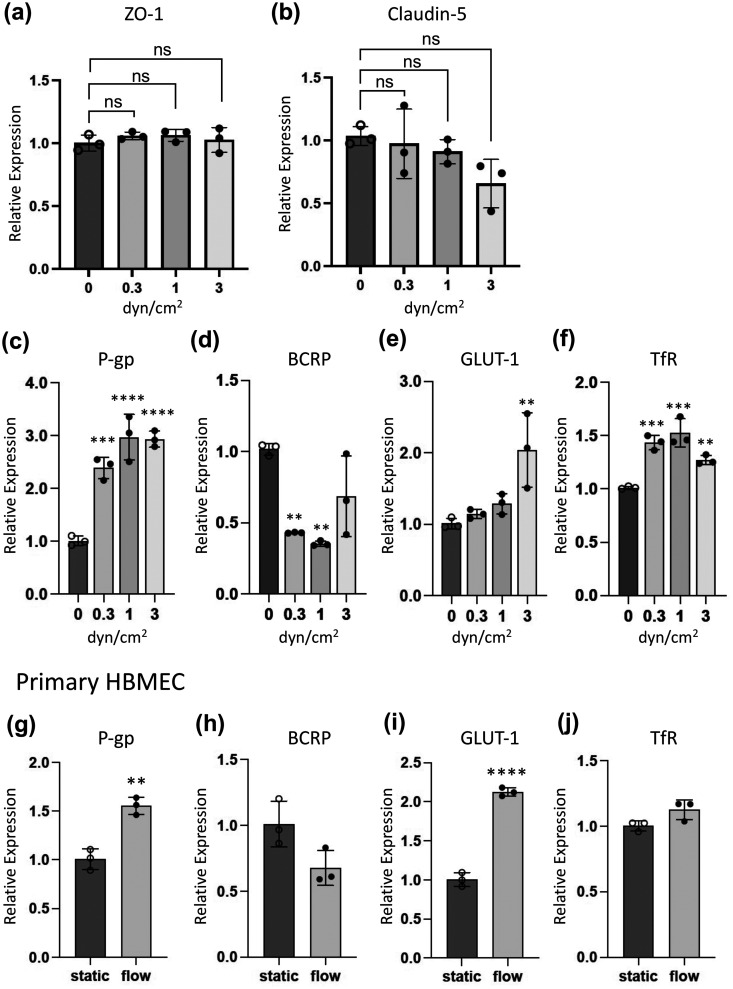
Quantification of BBB marker gene expression after perfusion culture of cells. HBMEC/ci18 cells were cultured in the device for 72 h under the static and flow (0.3, 1, and 3 dyn/cm^2^) conditions, and then expression levels of genes encoding tight junction protein (a) and (b) and membrane transporters/receptor (c)–(f) were analyzed by qPCR. Relative gene expression was determined by normalizing the expression of genes with that of *GAPDH*. Data are shown as the means ± S.D. (*n *=* *3). Significance was assessed by one-way ANOVA with Dunnett's multiple comparison test; ^*^P <* *0.05, ^*^^*^P <* *0.01, ^*^^*^^*^P <* *0.001, ^*^^*^^*^^*^P <* *0.0001. (g-j) Primary HBMECs were cultured in the device for 72 h under the static or flow (1 dyn/cm^2^) conditions, and then expression levels of various BBB membrane transporters/receptors (P-gp, BCRP, GLUT1, and TfR) were analyzed by qPCR. Relative gene expression was determined by normalizing the expression of genes with that of *GAPDH*. Data are shown as the means ± S.D. (*n *=* *3), and significance was assessed by unpaired two-tailed Student's *t*-test; ^*^^*^P <* *0.01, ^*^^*^^*^^*^P <* *0.0001.

To quantitatively evaluate the effects of FSS on the expression of BBB marker genes, real-time quantitative polymerase chain reaction (qPCR) analysis was performed using the mRNAs extracted from the HBMEC/ci18 cells cultured under the various flow conditions (0, 0.3, 1, and 3 dyn/cm^2^). As shown in Figs. 4(a) and 4(b), expression levels of genes encoding tight junction protein ZO-1 and Claudin-5 were not significantly affected under any FSS conditions tested. This result is consistent with the TEER measurement results demonstrated in Fig. 2. Regarding the TEER values and gene expression levels of tight junction proteins, FSS lower than 1 dyn/cm^2^ seems not to cause substantial loss of barrier tightness. Meanwhile, gene expression levels of P-gp and TfR were significantly elevated under the flow conditions by threefold and 1.5-fold, respectively. It is likely that expression levels of these genes are upregulated and reached a plateau even at the flow rate of 0.3 dyn/cm^2^ [Figs. 4(c) and 4(f)]. The gene expression level of GLUT-1 was not altered under the lower FSS conditions (0.3 and 1 dyn/cm^2^) but increased by 2.0-fold at the flow rate of 3 dyn/cm^2^. BCRP was significantly downregulated under the flow conditions (43.0% at 0.3 dyn/cm^2^; 35.4% at 1 dyn/cm^2^). These data demonstrated that our microfluidic system is capable of detecting the altered gene expression of BBB markers in response to the FSS and showed that relatively low range of FSS (0.3–1 dyn/cm^2^) is sufficient to induce the maximal effect of FSS on the gene expression of BBB markers.

Next, we cultured primary human microvascular endothelial cells in the device (1 dyn/cm^2^) and analyzed the gene expression of BBB markers to compare the FSS-responses of cells with those of the HBMEC/ci18 cells [Figs. 4(g)–4(j)]. The FSS upregulated P-gp (1.6-fold, *P *=* *0.0024) and GLUT1 (2.1-fold, *P *<* *0.0001) but had little effect on the expression of TfR (1.1-fold, P = 0.0660). Expression of BCRP was decreased to 68% of that of the static control, but the difference was marginally significant (*P *=* *0.0574). To greater or lesser degrees, both cell types exhibited basically similar responses to FSS, indicating that HBMEC/ci18 cells retain the basic ability of the human BBB endothelium to respond to FSS even after the process of cell immortalization.

### Evaluation of permeability barrier of the human BBB endothelium grown in the device

**FIG. 5.**
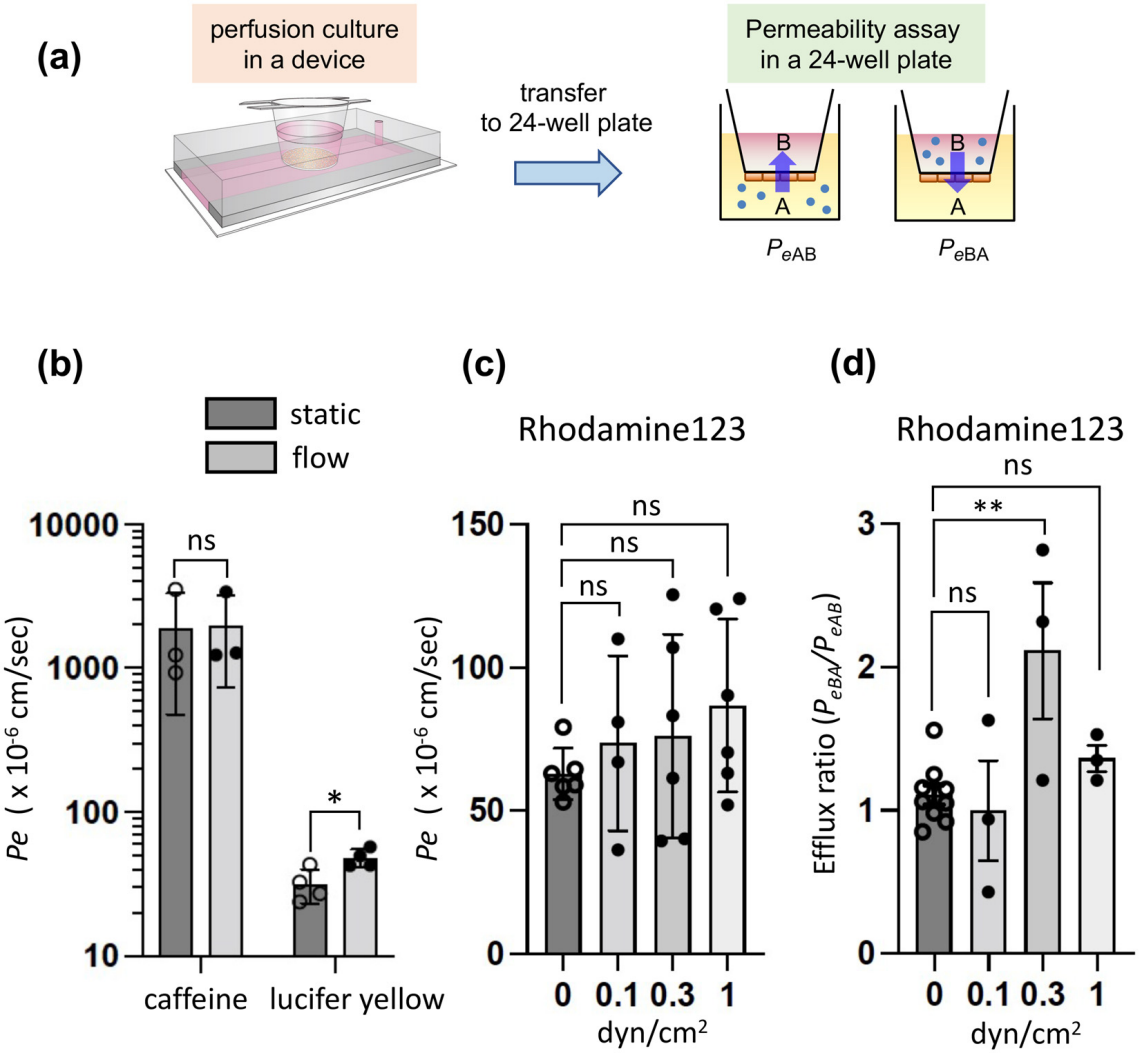
Evaluation of the barrier function of HBMEC/ci18 cells cultured in the device. (a) Schematic procedure for permeability test. HBMEC/ci18 cells were cultured in the device under the static and flow conditions for 72 h, and then the culture inserts were transferred to a 24-well plate to perform a permeability test of chemicals. Chemicals were added to the apical (well) or basolateral (insert) side of the endothelial cell barrier, and the assay buffer was collected from the basolateral or apical side to determine effective permeability coefficient (*Pe*). (b) Permeability of caffeine (5 *μ*M) and lucifer yellow (10 *μ*M). HBMEC/ci18 cells were cultured in the device under the static and flow (1 dyn/cm^2^) conditions, and *P*_eAB_ was calculated. Data represent the means ± S.D. (*n *=* *3 for caffeine; *n *=* *4 for lucifer yellow), and significance was assessed by unpaired two-tailed Student's *t*-test; *P <* *0.05. (c) and (d) Rhodamine 123 permeability under various flow conditions. Rhodamine 123 (5 *μ*M) was added in the apical or basolateral side of the cells. Efflux ratio was calculated based on the *P*_eAB_ and *P*_eBA_ to evaluate the efflux activity of P-gp. Data represent the means ± S.D. [*n *=* *6, 4, 6, and 6 for 0, 0.1, and 0.3, and 1 dyn/cm^2^ in panel (c); *n *=* *9, 3, 3, and 3 for 0, 0.1, 0.3, and 1 dyn/cm^2^ in panel (d), respectively]. Significance was assessed by one-way ANOVA with Dunnett's multiple comparison test; **P <* *0.01.

The advantage of our device is that BBB endothelium grown on the cell culture insert is detachable from the device after the perfusion culture of cells, and the permeability coefficient of the test compounds across the endothelium can be determined by the conventional transwell assay. First, we measured the permeability of caffeine^35^ (BBB permeable compound) and lucifer yellow (BBB non-permeable compound) using the BBB endothelium grown in the device. These two compounds were used as the reference set for establishment of dynamic range of permeability across the BBB endothelium. As shown in Fig. 5(a), a confluent HBMEC/ci18 cell layer was cultured in the device under the static or flow condition for 72 h, and then the culture inserts were carefully transferred to the 24-well culture plates to perform the permeability tests. Each reference compound was added to the apical compartments of the cells (the wells of the companion plate), and the effective permeability coefficients *P_e_* were determined as described in “Methods.” The *P_e_* value of caffeine was quite high and almost the same in both culture conditions (1900 ± 1400 × 10^−6 ^cm/s in the static culture; 2000 ± 1200 × 10^−6 ^cm/s in the perfusion culture at 1 dyn/cm^2^) [Fig. 5(b)]. In contrast, the *P_e_* values for lucifer yellow was much lower (32 ± 9 × 10^−6 ^cm/s in the static culture; 48 ± 7 × 10^−6 ^cm/s in the perfusion culture at the same flow rate) than those of the caffeine and slightly increased under the flow condition [Fig. 5(b)]. This difference of the *P_e_* values between caffeine and lucifer yellow indicates that the permeability barrier was successfully formed in the device, and the dynamic range was not markedly affected by the perfusion culture of cells.

### Functional analysis of drug efflux transporter

To further investigate the functionality of the BBB endothelial barrier in our device, we evaluated the drug efflux activity of P-gp, the most important multidrug efflux pump at the BBB. P-gp activity was assessed by bidirectional transport analysis using rhodamine 123 as a P-gp substrate. As shown in Fig. 5(c), the *P_eAB_* [*P_e_* for the apical (A)-to-basolateral (B) side direction] values for rhodamine 123 were unaffected under any FSS conditions tested (0.1–1 dyn/cm^2^) as compared to that of the static control (64 ± 9 × 10^−6 ^cm/s). Interestingly, the efflux ratio (ER) for rhodamine 123, which indicates the drug efflux activity determined by *P_eBA_/P_eAB_* (*P_e_* for the basolateral-to-apical direction is divided by the *P_e_* for the apical-to-basolateral direction), was significantly increased to 2.1 ± 0.8 under the FSS condition of 0.3 dyn/cm^2^, whereas ER for the static control was much lower (1.1 ± 0.2) [Fig. 5(d)]. These results suggest that P-gp efflux activity under the flow condition is 1.9-fold higher than that of the static control. This is probably due to the upregulation of the P-gp gene by FSS as shown in Figs. 3(c) and 4(c). Since the HBMEC/ci18 cell layer maintains the tightness of the barrier at 0.3 dyn/cm^2^ (Fig. 2), the upregulation of the P-gp gene seems to be directly linked to the increase in the functional efflux pump activity. Unexpectedly, despite the increased P-gp gene expression, the ER of rhodamine 123 was reduced to 1.4 ± 0.2 at 1 dyn/cm^2^. This reduction is convincing if the partial loss of TEER value and the slight increase in the *P_e_* value for lucifer yellow at 1 dyn/cm^2^ are taken into consideration [Figs. 2 and 5(b)]; the partial loss of barrier tightness is probably counteracting the effect of increased P-gp gene expression to some extent at the flow rate of 1 dyn/cm^2^.

As reported in the previous study,^17^ ER of rhodamine 123 for the conventional mono-culture and tri-culture model using the immortalized BBB cell lines (HBMEC/ci18, HBPC/ci37: pericytes, and HASTR/ci35: astrocytes) is approximately 1.3 and 1.7, respectively. Under the optimized flow condition (0.3 dyn/cm^2^), our device could achieve the higher ER value (2.1) without co-culturing the BBB-associated cells (pericytes and astrocytes) than that of the conventional tri-culture model (1.7). Although it remains unclear whether these models with the comparable ER values exhibit the similar BBB property in total, achievement of the higher ER values without the co-culture is one of the advantages of our device. For the future study, combination of our device with the tri-culture model is expected to realize more better system to investigate the bidirectional transport of drugs mediated by the BBB transporters/receptors in the FSS-stimulated BBB endothelium.

## CONCLUSION

This study demonstrated the utility of a microfluidic device to apply FSS on the BBB endothelium grown on the cell culture insert, which is designed to be detachable from the device after the perfusion culture of cells. This design makes it easier to investigate the bidirectional transport of drugs across the FSS-stimulated BBB endothelium. Researchers do not need the specific techniques and equipment to carry out the perfusion culture of cells, which are usually necessary to operate the BBB chips that mimic the physiological complexity and structure of human BBB. Using this simple and user-friendly microfluidic device with the HBMEC/ci18 cells, we successfully formed the BBB endothelial barrier without the substantial loss of barrier tightness under the relatively low flow conditions (0.1–1 dyn/cm^2^). Gene expression analysis showed that key BBB transporters and receptors including P-gp, GLUT1, TfR, but not BCRP in the HBMEC/ci18 cells were significantly upregulated in response to the FSS. Similar responses were observed in the primary HBMECs, indicating that our device with the immortalized HBMECs can mimic the FSS-responses of the primary human BBB endothelium. Finally, using the bidirectional transport assay of rhodamine 123 as a substrate for P-gp drug efflux pump, we showed that our device achieved 1.9-fold higher P-gp efflux activity under the optimized flow condition as compared to the static control. This is probably linked to the observation that the gene expression of P-gp was upregulated by 2.5-fold under the flow conditions. Thus, our device may serve as a useful platform that can provide the functional BBB endothelium with higher drug transporter activity even in the absence of BBB-associated cells.

Recently, there are increasing number of studies focusing on the drug delivery to the brain by utilizing the carrier protein-mediated transcellular transport or receptor-mediated transcytosis. At this point of view, the functional expression of GLUT1 and TfR is one of the critical properties of the BBB model. Although BBB-on-chip models are shown to express this kind of transporters/receptors, the complex structure and difficulty in operating the device, especially in 3D BBB models, make it hard to quantitatively analyze the directional transport of drugs with the validated transport specificity. Since GLUT1 and TfR were upregulated in our device and the FSS is expected to induce the polarized localization of certain types of membrane transporters,^10^ we believe that our device comprising the detachable cell culture insert will be advantageous to analyze the GLUT1- or TfR-mediated directional drug transport and its regulation in the FSS-stimulated BBB endothelium.

## METHODS

### Design and fabrication of the microfluidic device

The cell culture insert-integrated microfluidic device was fabricated as previously described with minor modification.^18^ Briefly, the device is simply composed of a microfluidic channel and a cylindrical hole for a 24-well culture insert with a collagen vitrigel membrane (ad-MED Vitrigel; a vitrified membrane composed of bovine native collagen type I^19^ with 10 *μ*m thickness and 0.33 *μ*m^2^ membrane area (ϕ = 3.2 mm), Kanto Chemical Co., Inc., Tokyo, Japan). The device is immobilized on a 50 mm deep petri dish, and the gap space between the device and dish is utilized as a medium reservoir. The cylindrical hole is located above the microchannel so that the perfusable microchannel is formed when the culture insert is placed in the hole. The cross-sectional size of the microfluidic channel is 7.8 mm (width) × 150 *μ*m (height). The microfluidic device was fabricated using PDMS (Sylgard 184 Silicone Elastomer, Dow Corning, Midland, MI). The resin molds for the microfluidic device were prepared using a commercial 3D printing machine (Agilista, Keyence Corp.). The PDMS elastomer, mixed at a 10:1 (w/w) ratio of cross-linking agent, was cast into the molds and solidified by heating (70 °C, 2 h). After making an outlet port using biopsy (φ = 1 mm), the top and bottom PDMS parts were assembled and bonded together using a water vapor plasma treatment (Aqua plasma, SAMCO). Finally, the device was immobilized on the 50 mm deep petri dish by solidifying PDMS as a glue. The PDMS device and tubes for perfusion culture (a polyvinyl chloride tube for a peristaltic pump and ethylene tetrafluoroethylene (ETFE) tubes to be connected to the device) were sterilized using 70% ethanol and UV/ozone gas sterilizer (CoolCLAVE Plus, Genantis, Inc.).

### Cell culture and flow experiments

HBMEC/ci18 (human brain microvascular endothelial cell/conditionally immortalized clone 18) is a temperature-sensitive immortalized human brain microvascular cell line, which has been established in the previous work.^17^ The cells were maintained on the type-I collagen-coated dishes in the EGM-2 growth medium (LONZA, Walkersville, MD) with 4 *μ*g/ml blasticidin S (Thermo Fisher Scientific, Waltham, MA) and penicillin (100 units/ml)−streptomycin (100 *μ*g/ml) (Gentamicin provided with EGM-2 BulletKit was not used in this study). The cells were grown to subconfluence at 33 °C in a humidified incubator with 5% CO_2_ and subcultured using 0.1% trypsin-ethylenediaminetetraacetic acid (EDTA) at a split ratio of 1:2. Primary human BMECs were purchased from Neuromics (Edina, MN), cultured in EGM-2 growth medium at 37 °C, and used for the experiments within three passages.

Before seeding HBMEC/ci18 onto the bottom side of the collagen vitrigel membrane of 24-well culture inserts, the bottom side of the membrane was incubated with 100 *μ*g/ml human type-IV collagen (Sigma, St Louis, MO)/100 *μ*g/ml human fibronectin (Sigma) solution at 37 °C for 30 min. After the incubation, the inserts were dried in air and rinsed once with phosphate-buffered saline (PBS). Subconfluent HBMEC/ci18 was harvested and resuspended in the EGM-2 growth medium without VEGF-A, EGF, and blasticidin S (VE-free EGM-2) at the density of 4.6 × 10^5^ cells/ml. The cell suspension (7 × 10^4^ cells/150 *μ*l for an insert) was then placed on the outer side of the membrane of the insert and incubated for 30 min to allow the cell adhesion. After 30 min of incubation, the culture inserts were placed in the 24-well companion plate with VE-free EGM-2 medium, and the cells were cultured overnight to form the confluent monolayer on the vitrigel membrane.

For the perfusion culture with the microfluidic device, the microfluidic channel and cylindrical hole were filled with VE-free/EGM-2 medium, and the cell-laden culture insert was carefully placed in the cylindrical hole of the device. A polyvinyl chloride tube for a peristaltic pump (MINIPULS3, Gilson, Inc., Middleton, WI) was connected to the ETFE tubes at both ends. After filling the tube with VE-free/EGM-2 medium, one side of the ETFE tube was connected to the hole made at the cover of culture dish via rubber sponge septum, and the other side of the ETFE tube was connected to the outlet port of the microchannel. This experimental setup was placed in the 33 °C CO_2_ incubator, and the perfusion culture was performed with 14 ml VE-free/EGM-2 medium for a device. The applied fluid shear stress *τ* was calculated based on the average flow speed *u* according to the following equation:^36^

$$\tau =\frac{6\mu }{h}u,$$

where *u* and *h* are the average speed of the culture medium and the height of the microfluidic channel, respectively. The viscosity *μ* was given as 8.9 × 10^−4 ^Pa s.

### Measurement of transendothelial electrical resistance (TEER)

TEER across the HBMEC/ci18 cell layer was evaluated after the 3 days culture in the device under static or flow condition. The culture inserts were dismounted from the device and placed to the wells of the 24-well companion plate with 1.4 ml culture media used in the devices. TEER was measured by using a Millicel ERS-2 Voltohmmeter (Millipore, Darmstadt, Germany) with STX01 electrode (Millipore). TEER values were determined as follows:

$$\mathrm{TEER}\left[\Omega \cdot {\text{cm}}^{2}\right]\;=\;\left(R_{\text{measure}}\left[\Omega \right]\;-R_{\text{blank}}\left[\Omega \right]\right)\cdot A\left[{\text{cm}}^{2}\right],$$

where *R*_measure_ is the measured electrical resistance across the cell layer on the collagen vitrigel membrane, *R*_blank_ is the electrical resistance of the vitrigel membrane only (without cells), and *A* is the surface area of the vitrigel membrane (0.33 cm^2^).

### Immunostaining

The cells cultured on the vitrigel membrane were rinsed with PBS and fixed with 4% paraformaldehyde in PBS (4% PFA/PBS) for 15 min at room temperature. After the fixation, cells were permeabilized with 0.2% Triton X-100 for 5 min (only for the immunostaining of CD31, ZO-1, and Transferrin receptor) and incubated with 5% bovine serum albumin (Sigma) in PBS for 20 min to block nonspecific binding of the antibody. These preparations were then incubated at 4 °C overnight with either of the primary antibodies: mouse anti-CD31 monoclonal antibody (1:200, R&D Systems, Minneapolis, MN), mouse anti-ZO-1 monoclonal antibody (1: 200, Thermo Fisher Scientific), mouse anti-P-gp monoclonal antibody (1:100, Santa Cruz Biotech, Santa Cruz, CA), anti-BCRP monoclonal antibody (Cell Signaling Technology, Danvers, MA), anti-Glut1 monoclonal antibody (1:200, Abcam, Cambridge, MA), anti-Transferrin Receptor monoclonal antibody (1:200, clone 13E4, Abcam), followed by 1 h incubation with Alexa Fluor 488- or, Alexa Fluor 568-conjugated anti-IgG secondary antibody (Thermo Fisher Scientific), and 1 *μ*g/ml of 4′, 6-diamidino-2-phenylindole (DAPI, Sigma) in 5% BSA/PBS. Stained samples were washed three times with PBS and post-fixed using 4%PFA/PBS for 20 min at room temperature. Cross-sectional or stacked images were acquired by confocal laser scanning microscopy LSM780 and ZEN imaging software (Carl Zeiss Microscopy, Oberkochen, Germany).

### Quantitative gene expression analysis

After culturing cells in the device, the culture inserts were dismounted from the device and rinsed once with cold PBS. Total RNA was prepared using RNeasy Plus Mini Kit (QIAGEN, Valencia, CA). First strand cDNA was synthesized from 100 to 200 ng of total RNA using a PrimeScript RT reagent Kit with gDNA Eraser (Takara Bio Inc, Otsu, Japan). Quantitative polymerase chain reaction (qPCR) was run on a StepOnePlus real-time PCR system (Thermo Fisher Scientific) in triplicate for each target genes using TB Green Premix Ex Taq II (Tli RNaseH Plus) according to the manufacturer's instructions. The relative gene expression was normalized to that of *glyceraldehyde 3-phosphate dehydrogenase* (*GAPDH*) and calculated using the comparative *Ct* method. Primer sequences for genes encoding ZO-1 (Gene name: *TJP1*), Claudin-5 (Gene name: *CLDN5*), P-gp (Gene name: *ABCB1*), BCRP (Gene name: *ABCG2*), GLUT-1 (Gene name: *SLC2A1*), and TfR (Gene name: *TFRC*), were obtained from the previous works.^17,37^

### Permeability assay

Permeability of test compounds across the confluent monolayer of HBMEC/ci18 cells was evaluated after 3 days culture in the device under static or flow condition. The culture inserts were dismounted from the device and placed in the wells of the 24-well companion plate. After the inserts were rinsed twice with Hank's balanced salt solution with calcium and magnesium (HBSS (+), Nacalai Teskque, Kyoto, Japan), the medium was replaced with 300 *μ*l HBSS (+) in the basolateral compartment (insert) and 1400 *μ*l HBSS (+) in the apical compartment (well), followed by preincubation at 33 °C for 30 min in a CO_2_ incubator. Permeability test was performed as previously reported.^17^ Briefly, each test compound, including caffeine (5 *μ*M, Sigma), lucifer yellow (10 *μ*M, FUJIFILM Wako Pure Chemicals, Osaka, Japan), and rhodamine 123 (5 *μ*M, FUJIFILM Wako Pure Chemicals), was added to the apical compartment and incubated at 33 °C in the CO_2_ incubator. At the sampling time points (30, 60, and 90 min for caffeine and lucifer yellow; 20, 40, and 60 min for rhodamine 123), the medium (20 *μ*l) was collected from the basolateral compartment, and the same volume of HBSS (+) was added to the basolateral compartment for each sampling to keep the medium volume constant. To quantify the lucifer yellow or rhodamine 123 in the collected medium, the fluorescence intensity of the sample solution was measured by Cytation 5 Cell Imaging Multi-Mode Reader (Bio Tek Instruments, Winooski, VT) with wavelength ex/em = 428/536 nm for lucifer yellow, and 505/534 nm for rhodamine 123. Using calibration curves, concentrations of each test compound in the samples were determined, and the effective permeability coefficient (*P_e_*) was calculated as described previously.^17^ Caffeine was quantified using Caffeine ELISA Kit (BioVision, Mountain View, CA) according to the manufacturer's instructions. For a bidirectional permeability assay, rhodamine 123 was added to the apical (A) or basolateral (B) side, and the medium was collected from the (B) or (A) side, respectively. *P_e_* for the apical-to-basolateral (*P_eAB_*) and the basolateral-to-apical (*P_eBA_*) directions were calculated, and the efflux ratio was determined by *P_eBA_/P_eAB_.*

## SUPPLEMENTARY MATERIAL

See the supplementary material for figures regarding the setup of the perfusion culture system, flow simulation data, and method for flow simulation.

## Data Availability

The data that support the findings of this study are available from the corresponding author upon reasonable request.
